# Thermal and Nuclear Quantum Effects at the Antiferroelectric to Paraelectric Phase Transition in KOH and KOD Crystals

**DOI:** 10.1021/acs.jpcc.1c06953

**Published:** 2021-09-24

**Authors:** Erika Fallacara, Philippe Depondt, Simon Huppert, Michele Ceotto, Fabio Finocchi

**Affiliations:** † Sorbonne Université, CNRS, Institut des NanoSciences de Paris (INSP), 4 Place Jussieu, Paris F-75005, France; ‡ Dipartimento di Chimica, Università Degli Studi di Milano, Via Golgi 19, Milano 20133, Italy

## Abstract

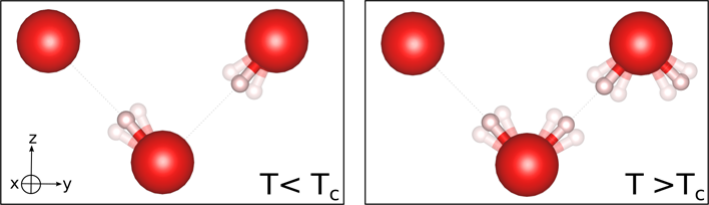

Crystalline KOH undergoes an antiferroelectric (AFE) proton ordering phase transition at low temperatures, which results in a monoclinic bilayer structure held together by a network of weak hydrogen bonds (HBs). The Curie temperature shifts up when the compound is deuterated, an effect that classical MD is not able to catch. For deeper insights into the transition mechanism, we carry out *ab initio* MD simulations of KOH and KOD crystals by including quantum effects on the nuclei through Feynman path integrals. The geometric isotope effect and the evolution of the lattice parameters with temperature agree with the experimental data, while the purely classical description is not appropriate. Our results show that deuteration strengthens the HBs in the low-*T* AFE ordered phase. The transition is characterized by the flipping of OH/OD groups along a bending mode. Above the transition, the system is driven into a dynamical disordered paraelectric phase.

## Introduction

Since its discovery in 1920 by Valasek,^1^ ferroelectricity has been a focus of intense research addressed by both academia and industries. Considerable interest has been devoted to hydrogen-bonded ferroelectrics (FEs) and antiferroelectrics (AFEs) for their functionalization in nonlinear optics.^2^ The prototype material is potassium dihydrogen phosphate (KDP) discovered in 1935,^3^ where strong hydrogen bonds (HBs) link the tetrahedral PO_4_ units. In the low-*T* FE phase, the protons are off-center ordered in the HBs, which are nearly perpendicular to the FE *c*-axis.^4^ KDP becomes paraelectric (PE) at higher temperatures.^5,6^ The proton off-centering stretches the O–O distance and thus correlates to the lattice expansion and heavy-ion distortions.

The ≃100 K increase of the Curie temperature upon deuteration has been attributed to the depletion of hydrogen tunneling between the two off-center positions and the weakening of the HBs associated with the FE distortion.^7^ The weakening of strong HBs (O–O distance between 2.45 and 2.7 Å) is the well-known Ubbelohde effect.^8^ Indeed, deuteration produces an increase in the bond length of ≃0.03 Å and consequently makes the short bonds weaker. A clear example is provided by the ice VII to ice X transition under pressure,^9,10^ where O–H···O bonds become centered and thus stronger at lower pressures than O–D···O bonds. The KDP paradigm shows that the detailed characteristics of HBs, such as their length and strength, greatly affect the behavior of the protons and the dielectric properties of hydrogen-bonded crystals. The quantum nature of light nuclei, such as H and D, can also play a crucial role and give rise to spectacular isotope effects.

**Figure 1:**
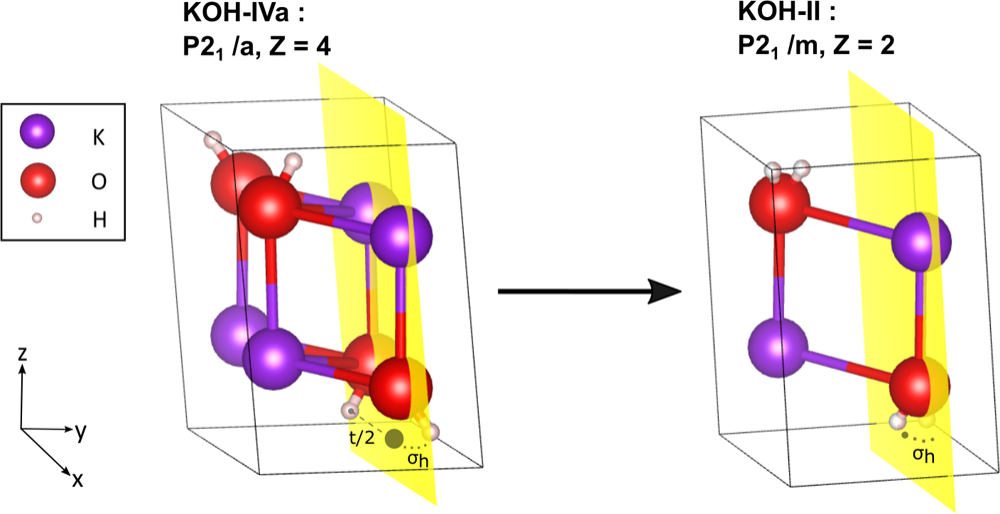
Sketch of the unit cell of KOH-IVa and KOH-II from neutron powder diffraction data.^17^ Ionic radii are represented according to studies by Shannon.^31^

In this regard, alkali hydroxides AOX (A = Li to Cs, X = H, D) display stretched and weak HBs. At ambient pressure, proton ordering phase transitions occur in all the alkali hydroxides with the exception of LiOH/LiOD and NaOH.^11−25^ The critical transition temperature, *T*_c_, is between 150 and 310 K, and a conventional isotope effect *T*_c_(AOD) > *T*_c_(AOH) of about 30 K is observed for K, Rb, and Cs. Furthermore, pressure can promote similar transitions even when these are forbidden at ambient conditions.^26−30^ Thus, the phase diagram of AOX crystals is determined by a complex interplay between the temperature, the metal cation A^+^, the hydrogen isotope X, and the pressure. In the following sections, we focus on the phase transition in K hydroxides, which has been characterized as a transformation from an ordered low-*T* AFE phase (phase IVa) to a disordered high-*T* PE phase (phase II). Both crystals are monoclinic. KOH-IVa has a *P*2_1_/*a* space group and four molecular units per cell, while KOH-II ha as *P*2_1_/*m* space group and two molecular units per cell (Figure 1).

The crystallographic positions corresponding to the hydrogen atoms in the II phase have been determined, as macroscopic averages, at half-occupancy over the two Wyckoff sites 4*f* related by the mirror plane. This has been interpreted within a model of dynamically disordered dipoles in which the hydrogen atoms are rapidly hopping across the mirror planes *y* = ±1/4. However, the detailed atomic-scale structure still lacks a careful description. Another issue concerns *T*_c_, which is sensitive to the isotope substitution: *T*_c_ is 233 K in KOH and 257 K in KOD.^17^ The larger Curie temperature *T*_c_ upon H → D substitution suggests that nuclear quantum effects (NQEs) are important to unravel the mechanism of the transition. However, the failure of classical statistical mechanics to account for isotope effects makes the understanding of those phase transitions still an open question. In this article, we stress the importance of NQEs on the phase stability of KOH and KOD crystals as a reference example for the AOX family and elucidate the mechanism of the phase transition as well as the nature of the isotope effect.

## Computational Details

For an accurate and appropriate description beyond the classical picture, we account for the nuclear degrees of freedom *via* path-integral (PI) MD.^32,33^ The electronic structure and atomic forces were calculated by using the DFT^34^ within the PBE approximation^35^ by using the quantum espresso (QE) package.^36^ Phonon calculations were performed within the harmonic approximation *via* the density functional perturbation theory (DFPT).^37^ We employed ultrasoft pseudopotentials for the oxygen and hydrogen atoms and norm-conserving pseudopotentials for the potassium atom. The plane-wave expansion cutoff energies were 50 Ry for the Kohn–Sham states and 8 times as large for the charge density and the potential. The unit cell contains 4 formula units, and the corresponding Brillouin zone was sampled using a 2 × 4 × 3 Monkhorst–Pack grid.

The potential energy surface (PES) at *T* = 0 K was reconstructed according to the following procedure. We performed several climbing image-nudged elastic band (CI-NEB)^38^ calculations at fixed lattice constants by varying the initial and the final configurations. All the initial and final configurations have been obtained by varying the crystal *y*-component of the OH vector that is associated with the coordinate of reaction of the phase transition, as *y*′_OH_ = *y*_OH,eq_ × α, in which *y*_OH,eq_ is the equilibrium *y*-component of the OH vector and α is a coefficient in the range [−1.4, 1.4]. In the CI-NEB input, the initial and final configurations were provided with fixed *y* crystal positions of oxygen and hydrogen atoms and, in some cases, also the ones of potassium atoms. The initial and final configurations were free to relax under the above-mentioned constraints. In this way, we obtained several minimum energy paths (MEPs) that sample the overall PES and an estimate of the classical energy barrier at zero temperature.

The MD and PIMD simulations were carried out in the *NVT* canonical ensemble within a generalized Langevin equation at *T* = 77, 215, and 350 K. We employed the i-PI interface^33^ to the QE package. We used the PILE-L thermostatting scheme,^39^ with a centroid friction coefficient of 10 THz and the number of beads set as 32, which ensures the convergence of the kinetic and potential energies. At each temperature, the optimized crystal structures were obtained through systematic volume relaxation by gradually varying the lattice constants *a*, *b*, and *c* and the monoclinic angle β until the hydrostatic pressure was reached, in trajectories of 5 ps each, within an error on the average stress tensor components lower than 2 kbar. Finally, statistical averages were obtained from trajectories of duration time ranging from 20 to 40 ps.

## Results and Discussion

### Crystal Structure

**Figure 2:**
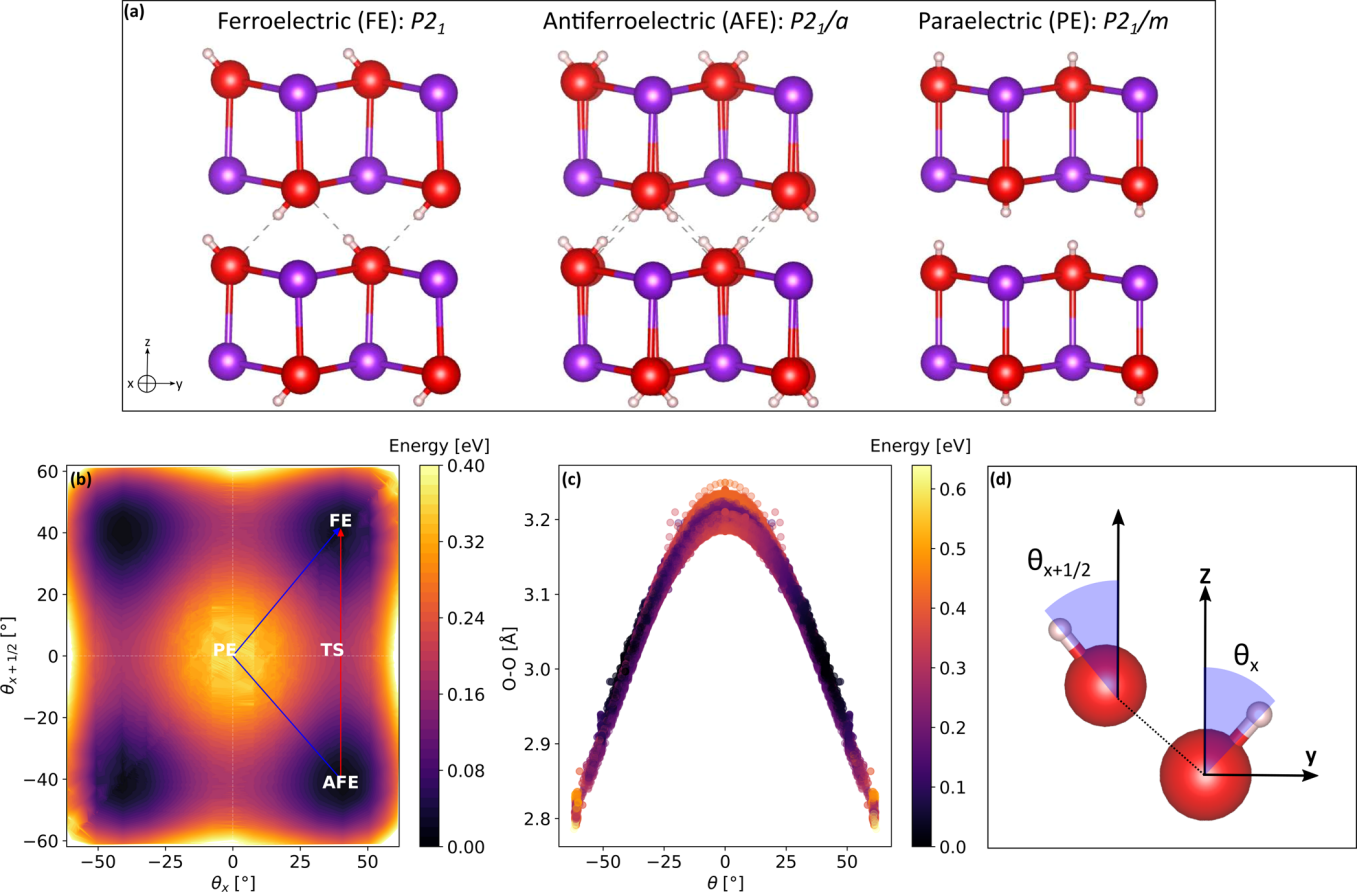
(a) *Static* FE, PE, and AFE structures of monoclinic KOH. (b) Classical PES (*cl*-PES) as computed at constant lattice parameters (see the lattice parameter for AFE in Table 1). (c) Correlation between the angle θ and the O–O distance along the *cl*-PES. The color bar corresponds to the potential energy. (d) Sketch of the order parameter θ.

As a first step into the crystal structure determination of monoclinic KOH and the description of the HBs, we performed classical geometry optimization at *T* = 0 K with initial constraints on the positions of the H atoms and identified the following reference *static* configurations: FE, AFE, and PE. While sharing a similar bilayered structure along the *z*-axis, the *static* configurations mainly differ in terms of the positions of the hydrogen atoms (Figure 2a).

FE and AFE configurations have permanent microscopic dipoles with a component along the *y*-axis. The neighboring layers are connected by weak HBs that contribute to the cohesion between the KOH bilayers. The AFE configuration has inversion symmetry and therefore does not bring any macroscopic dipole, in contrast to the FE configuration, where all hydroxyl groups are parallel. In PE configuration, the hydroxyl groups lie in a plane normal to the *y*-axis, in the Wyckoff site 2*e*, at crystal positions *y* = ±1/4.

As regarding the structural properties, the main differences between the *static* configurations are over *c* and β lattice parameters (see Table 1). For the purpose of the discussion, it is convenient to introduce the projection of *c* along the *z*-axis, that is, *c* sin β. This represents the interlayer distance, which is in turn correlated to the O–O distance and therefore characterizes the HB strength. The *static* AFE phase (*c* sin β = 5.302 Å) does not reproduce the measured interlayer distance *c* sin β in KOH-IVa, even when including semiempirical dispersion corrections (*c* sin β = 5.054 Å including Grimme D2^40,41^ and *c* sin β = 5.160 Å including Grimme D3^42^). The FE phase presents the same interlayer distance as AFE, while the KOH lattice is significantly expanded along the *z*-axis in PE with *c* sin β increasing up to 5.663 Å. This expansion is mainly due to the breaking of the interlayer HBs that correlates to the O–O distance, which increases from 3.00 Å in FE and AFE to 3.48 Å in PE.

**Table 1:** Lattice Parameters and HB Geometrical Parameters of Monoclinic Potassium Hydroxide from *Ab Initio* Calculations at *T* = 0 K and from Classical MD (*cl*-KOH/D) and PIMD Simulations (*q*-KOH and *q*-KOD), Compared with the Available Experimental Data^a^

	Crystallographic data						
		*a*	*b*	*c*	β	*c* sin β	O–O
Experimental data phase IVa: *P*2_1_/*a*, Z = 4							
100 K	KOD^18^	7.922	3.942	5.903	113.95	5.395	3.24
77 K	KOH^17^	7.892	3.945	5.947	114.24	5.423	(not reported)
Experimental data phase II: *P*2_1_/*m*, Z = 2							
300 K	KOD^18^	3.965	3.999	5.728	104.23	5.552	3.45
293 K	KOH^17^	3.951	3.999	5.750	103.58	5.589	3.33
	Theory (this work)						
0 K	FE	3.982	4.009	5.568	107.674	5.305	3.00
	AFE	7.958	4.014	5.770	113.230	5.302	3.00
	PE	3.963	4.028	5.782	101.634	5.663	3.48
77 K	*cl*-KOH/D	8.01	4.01	5.87	114.02	5.36	3.06
	*q*-KOD	8.04	4.02	5.98	114.25	5.45	3.18
	*q*-KOH	8.04	4.03	6.00	114.25	5.47	3.22
215 K	*cl*-KOH/D	7.98	4.05	5.76	110.00	5.41	3.09
	*q*-KOD	8.02	4.05	5.86	111.00	5.47	3.15
	*q*-KOH	8.02	4.06	5.88	111.00	5.49	3.18
350 K	*cl*-KOH/D	8.10	4.07	5.74	104.25	5.56	3.35
	*q*-KOD	8.15	4.08	5.77	104.25	5.59	3.38
	*q*-KOH	8.15	4.08	5.77	104.25	5.59	3.39

a In all cases, we computed *c* sin β that represents the projection of the *c*-axis along *z*. The reported experimental lattice parameters for the IVa phase are presented here after a change of basis. Lengths are reported in Å and angles in degrees.

### Classical Potential Energy Surface

For the purpose of understanding the atomic-scale transformations that occur in the crystal at finite temperature, first, we conducted extensive CI-NEB runs by following the procedure discussed in Computational Details. We explored several MEPs for the transitions AFE ↔ PE, PE ↔ FE, and AFE ↔ FE. These calculations provide a quite detailed picture of the *cl*-PES at *T* = 0 K that can serve as a reference when introducing thermal and nuclear quantum effects (Figure 2b). FE and AFE correspond to local minima on the *cl*-PES with the AFE being slightly more stable than the FE by 3 meV, mainly because of the larger repulsion between the protons in the FE phase. The *cl*-PES displays another notable configuration: the transition state (TS) along the AFE ↔ FE path. The TS is a saddle point whose total energy is ≃0.18 eV above the AFE’s most stable minimum. In the TS configuration, while the hydroxyl groups at the crystal *x* position are practically clamped at their equilibrium positions in the FE or AFE configurations, the other at *x* + 1/2 are very close to *y* = 1/4, which represents the mirror plane as in the PE configuration. Interestingly, the total energy of the TS configuration is nearly half that of the PE configuration (*E*_PE_ = 0.34 eV). We, therefore, set the *T* = 0 K classical barrier for flipping an entire row of hydroxyl groups across the mirror plane to the ≃0.09 eV/OH group. Such a barrier corresponds to a rather high temperature of about 1000 K. We point out that the topology of the *cl*-PES is consistent with OH groups that belong to the same chain of HBs (same crystal position *x*) flipping coherently back and forth. However, the system should not pass through the static PE configuration, which is a local maximum. Moreover, as discussed in the following section, the flipping process can be conveniently represented through the choice of the angle θ (Figure 2d). For instance, θ = 0 for all the hydroxyl groups in the static PE configuration, while the same value is only for half of them in the TS.

### Microscopic Order Parameter in the Phase Transition

**Figure 3:**
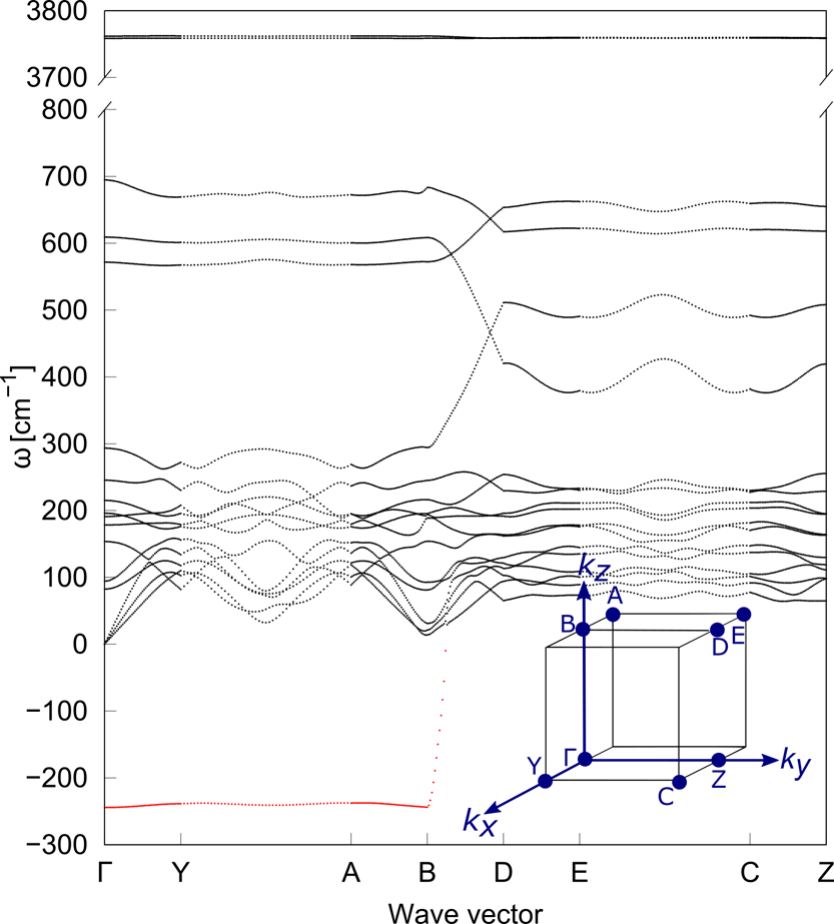
Phonon dispersion curves for the *static* PE phase of monoclinic potassium hydroxide, whose unit cell contains two formula units (*Z* = 2). The red branch corresponds to the unstable O–H libration mode. Imaginary frequencies are plotted as negative.

Consistently with the fact that the *static* PE configuration is not a local minimum, dynamical matrix calculations yield imaginary frequencies, revealing phonon instability for a mode corresponding to an O–H libration parallel to the *y*-axis direction. The phonon dispersion curves as computed in a large portion of the Brillouin zone (Figure 3) reveal that the O–H libration has an imaginary frequency along the symmetry lines Γ-Y-A-B (with *k*_*y*_ = 0) and a real one along the B-D-E-C-Z (with *k*_*y*_ = π/*b*) paths. In real space, the instability is associated with in-phase collective displacements of the hydroxyl groups parallel to the *y*-axis, which drive the *static* PE toward the AFE or FE configurations. According to the Landau description of phase transitions, a logical choice of the order parameter is the OH bending angle θ, defined as arctan(θ) = OH_*y*_/|OH_*z*_| where OH_*y*_ and OH_z_ are the *y* and *z* Cartesian coordinates of the OH displacement vector, respectively. This parameter is fully consistent with the *cl*-PES. The ordered phase in the Landau picture corresponds to ⟨θ⟩ = 0. An ambiguity could arise when considering the symmetry-broken phases that are either FE (same θ on parallel chains and ⟨θ⟩ ≠ 0) or AFE (opposite θ on parallel chains and ⟨θ⟩ = 0). In the following section, we specify the interchain correlation function that distinguishes the AFE from the FE configurations.

### Thermal and Nuclear Quantum Effects

As discussed previously, an important structural parameter is the interlayer distance *c* sin β. The IVa → II phase transition causes a thermal expansion of both KOH and KOD lattices with *c* sin β increasing by about 3% (see Table 1). *c* sin β is tightly correlated with the interlayer O–O distance and thus to the length of the HBs. The introduction of thermal and quantum effects provides for the IVa phase a better agreement with the experimental data (see Table 1, *c* sin β at 77 K). The high-*T* II phase cannot be represented *via* the *static* PE phase, which is unstable against vibrations along the *y*-axis direction. Nonetheless, we anticipate that classical MD provides good agreement with the experimental data at high temperatures (see Table 1, *c* sin β at 350 K). Isotope effects are indeed modest at 350 K, and thermal effects are mainly responsible for the microscopic configuration of potassium hydroxide at room temperature.

**Figure 4:**
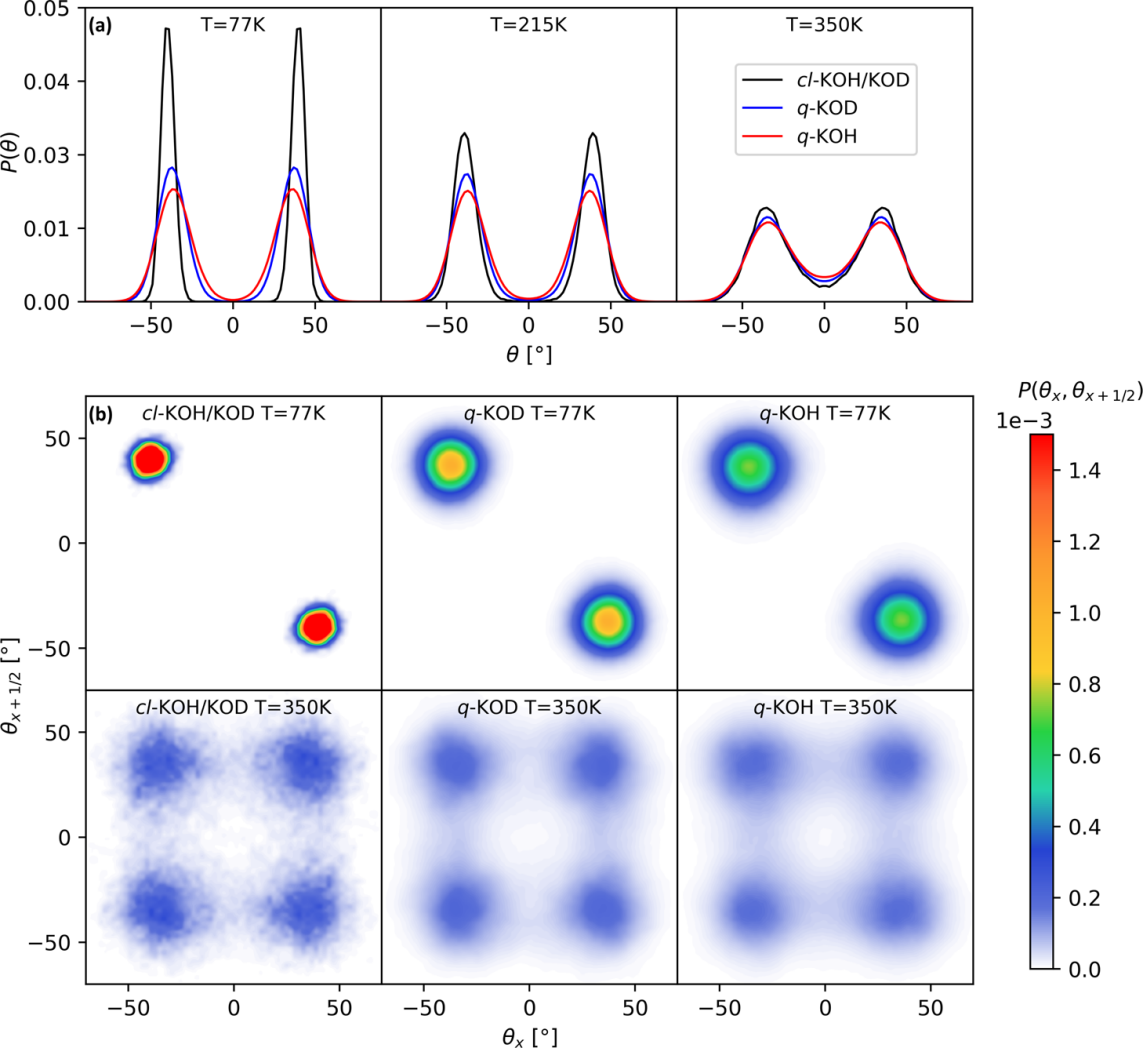
(a) Probability distribution for the microscopic order parameter θ and (b) joint probability distribution for θ_*x*_ and θ_*x*+1/2_ from *ab initio* classical MD (*cl*-KOH/D) and PIMD simulations (*q*-KOH and *q*-KOD). The subscripts *x* and *x* + 1/2 indicate the position of consecutive HB chains along the *x*-axis direction. The distributions have been symmetrized.

The probability distribution of the order parameter, *P*(θ), has a characteristic double–peak profile (see Figure 4a). At 77 K, the two maxima are located at θ ≃ ±40° and correspond either to clockwise (positive) or anticlockwise (negative) orientations of the OH and OD dipoles, resulting in HB chain polarized ∥±*y*. We can discriminate between the FE and AFE arrangements by looking at the correlation between the polar angles on adjacent HB chains along the *x*-axis direction, ⟨*θ*_*x*_, θ_*x*+1/2_⟩, *via* the joint probability distribution *P*(θ_*x*_, θ_*x*+1/2_) (Figure 4b). Positive and negative correlations denote parallel (FE) and antiparallel (AFE) configurations, respectively. As far as the AFE–FE potential energy barrier is concerned, we do not have a direct estimate when taking into account NQEs at 77 K. Nevertheless, it should lie between the classical barrier at *T* = 0 K (*i.e.*, 90 meV) and the quantum one at *T* = 350 K (Δ*F* = 18 meV), thus preventing flipping of the H/D atoms within our simulation time at *T* = 77 K. Indeed, dipole orientation-switching events are rare events at this temperature (*k*_B_*T* = 6.6 meV). By selecting distinct initial conditions for FE and AFE configurations, we computed the mean energy at 77 K along PIMD simulations for the KOH crystal. The AFE minimum is slightly more stable than the FE by around 5 meV, in line with the *static* calculations that yield the AFE more stable than the FE by 3 meV. All the previous findings are in agreement with experimental results that show an AFE order at low temperatures.^17,18^ At 215 K, the free energy barrier for the dipole switching is decreased, allowing the formation of both AFE and FE local arrangements, and a small number of flips occur within the time scale of our simulation. At 350 K, there is a rapid flipping of the dipoles, causing a continuous breaking and formation of the HBs, for both KOH and KOD. There are four possible degenerate states and none of these states coincides with the *static* PE configuration θ_*x*_ = θ_*x*+1/2_ = 0. Macroscopically, this results in a dynamical PE phase, with extremely tiny isotope effects and a half occupancy of the H/D 4*f* positions as suggested by the neutron diffraction experiments.^17^

### Behavior of the HBs

**Figure 5:**
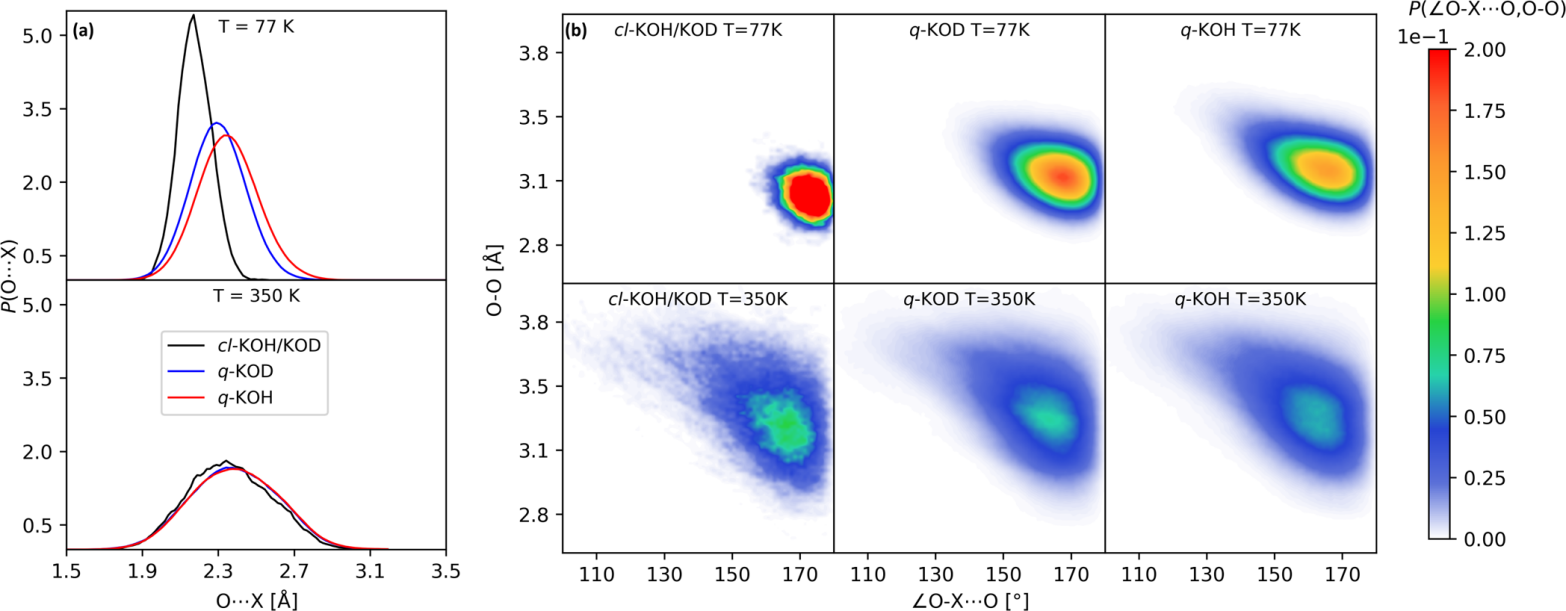
(a) Probability distribution for the O···X length and (b) joint probability distribution for the ∠O–X···O angle and the O–O distance from *ab initio* classical MD (*cl*-KOH/D) and PIMD simulations (*q*-KOH and *q*-KOD).

Ultimately, the phase transition in KOH and KOD is governed by the HBs. It is therefore of primary importance to analyze their behavior in temperature and how they are impacted by nuclear quantum effects in comparison to other systems. First, we remind that there is quite a strong correlation between the O–O interlayer distance (hence the length of the hydrogen bonds) and the bending angle θ (see Figure 2c): long and weak interlayer HBs favor small bending angles even in the classical *T* = 0 K picture and increase *c* sin β accordingly. Nuclear quantum effects conspire to make HBs in KOH and KOD even weaker, as shown by the probability distribution of the HB lengths, *P*(O···X), as shown in Figure 5a.

At 77 K, the quantum *P*(O···D) is peaked at 2.17 Å and the *P*(O···H) at 2.21 Å; classical MD yields much shorter O···H distances (2.07 Å). The distribution *P*(∠OXO, OO) of the ∠O–X···O angle, as shown in Figure 5b as a function of the O–O distance, clearly shows that in the quantum framework, HBs depart from linearity (∠O–X···O = 180°) significantly even at 77 K; ∠O–X···O angles below 160° are correlated to large O–O distances: the corresponding HBs are thus much more stretched than quasi-linear ones.

The previous effects are a direct consequence of the large angular quantum fluctuations on the proton, which stretch HBs and weaken them significantly with respect to the classical frame. Quantum fluctuations at low temperatures are slightly milder upon deuteration, as they can be seen by comparing the *P*(∠OXO, OO) distributions of KOD and KOH at 77 K (Figure 5b). Therefore, the behavior of HBs in potassium hydroxide (and the interlayer distance) is opposite to the conventional geometric isotope effect as introduced by Ubbelohde.^8^ Such a behavior can be rationalized in terms of competing effects;^43^ the zero-point motion along the O–H (O–D) stretching mode strengthens the HB. In contrast, fluctuations normal to the bond (thus on OH or OD bending modes) weaken it considerably. This latter effect dominates in weak HBs and specifically characterizes alkali hydroxides, where the H → D substitution leads to a contraction of HB lengths.^44^ As far as purely thermal effects are concerned, they globally destabilize HBs in potassium hydroxide, whether they involve protons or deuterons.

The classical MD *P*(O···X) distribution changes dramatically with temperature; it becomes much broader and its maximum shifts toward larger values. The classical *P*(∠OXO, OO) consistently shows larger angular fluctuations and O–O distances. At 350 K, *P*(O···X) shows a broad peak for the HB length, which is centered at 2.38 Å for KOH and 2.34 Å for KOD, with the classical peak very close to this value. The difference between KOH and KOD is negligible at this temperature, which is consistent with the fact that they share the same interlayer distance. At 350 K, the region ∠O–X···O < 130° is populated, which corresponds to an O–O distance >3.4 Å, a value for which the HBs are almost broken.

## Conclusions

KOH/KOD provides an interesting example in which nuclear quantum effects are strongly connected with other properties. NQEs impact the interlayer distance because HBs are weak and in turn a cascade of other effects. This apparent complexity can be untangled in a rather straightforward manner. We propose an atomic-scale model for the low-*T* IVa → high-*T* II transformation of KOH and KOD crystals, including both thermal and nuclear quantum effects. To summarize, in the low-*T* IVa phase, the positions of hydrogen atoms are alternately tilted in the *b* direction and correlated in the *a* direction in an AFE arrangement. On the other hand, the high-*T* II phase is characterized by an uncorrelated motion of the hydrogen atoms and, consequently, a dynamical disorder of FE–AFE states, that is, a dynamical PE phase with half occupancy of H and D sites. We thus confirm the order–disorder phase transition as originally suggested by Bastow *et al.* and illustrated by the large entropy contribution to the free energy. The transition temperature shift of 24 K upon deuteration, as observed experimentally, is a clear sign that NQEs contribute significantly to the transition mechanism. Both thermal and quantum zero-point fluctuations, which are larger in KOH than in KOD, are indeed responsible for the flipping back and forth of the H and D atoms and the two possible orientations of the OH/OD dipoles along a zig-zag chain of HBs between the layers. The transition also involves a large increase of the interlayer distance in the *c* direction. This interlayer region is the locus of weak HBs, a specific feature that distinguishes KOH/KOD crystals and most alkali hydroxides from other HB-based FEs such as KDP, which are characterized instead by strong HBs. KOH/KOD is highly impacted by quantum fluctuations, which further weaken the HBs and are at the origin of an inverse Ubbelohde effect, as observed at low temperatures (O···D shorter and stronger than O···H). This leads to an appreciable difference in the lattice parameters *c* and β for the two isotopologues: in KOD the bilayers are closer. To conclude, it is striking that a so simple system will exhibit such strong quantum-driven behavior at ambient pressure and such an easily accessible temperature.
